# Redundant roles of the phosphatidate phosphatase family in triacylglycerol synthesis in human adipocytes

**DOI:** 10.1007/s00125-016-4018-0

**Published:** 2016-06-25

**Authors:** Ana Temprano, Hiroshi Sembongi, Gil-Soo Han, David Sebastián, Jordi Capellades, Cristóbal Moreno, Juan Guardiola, Martin Wabitsch, Cristóbal Richart, Oscar Yanes, Antonio Zorzano, George M. Carman, Symeon Siniossoglou, Merce Miranda

**Affiliations:** 1 Joan XXIII University Hospital, Pere Virgili Health Research Institut (IISPV), Modular Building, C/ Mallafre Guasch, Tarragona, 43005 Spain; 2 Department of Biochemistry and Molecular Biology, Rovira i Virgili University, Tarragona, Spain; 3 Cambridge Institute for Medical Research, University of Cambridge, Wellcome Trust/Medical Research Council Building, Hills Road, Cambridge, CB2 0XY UK; 4 Chesterford Research Park, Little Chesterford, Saffron Walden, UK; 5 Department of Food Science and the Rutgers Center for Lipid Research, New Jersey Institute for Food, Nutrition and Health, Rutgers University, New Brunswick, NJ USA; 6 Institute for Research in Biomedicine (IRB Barcelona), The Barcelona Institute of Science and Technology, Barcelona, Spain; 7 Department of Biochemistry and Molecular Biology, Faculty of Biology, University of Barcelona, Barcelona, Spain; 8 Biomedical Research Networking Centre in Diabetes and Associated Metabolic Disorders (CIBERDEM), Instituto de Salud Carlos III, Madrid, Spain, http://www.ciberdem.org/; 9 Centre for Omic Sciences, Rovira i Virgili University, Reus, Spain; 10 Department of Pulmonary, Critical Care and Sleep Medicine, University of Louisville, Louisville, KY USA; 11 Division of Paediatric Endocrinology and Diabetes, Interdisciplinary Obesity Clinic, University Clinic for Child and Adolescent Medicine, University of Ulm, Ulm, Germany; 12 GEMMAIR Research Group – Applied Medicine, Department of Medicine and Surgery, Rovira i Virgili University (URV), Tarragona, Spain; 13 Department of Electronic Engineering, Rovira i Virgili University, Tarragona, Spain

**Keywords:** Basic science, Cell lines, Human, Lipid metabolism

## Abstract

**Aims/hypothesis:**

In mammals, the evolutionary conserved family of Mg^2+^-dependent phosphatidate phosphatases (PAP1), involved in phospholipid and triacylglycerol synthesis, consists of lipin-1, lipin-2 and lipin-3. While mutations in the murine *Lpin1* gene cause lipodystrophy and its knockdown in mouse 3T3-L1 cells impairs adipogenesis, deleterious mutations of human *LPIN1* do not affect adipose tissue distribution. However, reduced *LPIN1* and PAP1 activity has been described in participants with type 2 diabetes. We aimed to characterise the roles of all lipin family members in human adipose tissue and adipogenesis.

**Methods:**

The expression of the lipin family was analysed in adipose tissue in a cross-sectional study. Moreover, the effects of lipin small interfering RNA (siRNA)-mediated depletion on in vitro human adipogenesis were assessed.

**Results:**

Adipose tissue gene expression of the lipin family is altered in type 2 diabetes. Depletion of every lipin family member in a human Simpson–Golabi–Behmel syndrome (SGBS) pre-adipocyte cell line, alters expression levels of adipogenic transcription factors and lipid biosynthesis genes in early stages of differentiation. Lipin-1 knockdown alone causes a 95% depletion of PAP1 activity. Despite the reduced PAP1 activity and alterations in early adipogenesis, lipin-silenced cells differentiate and accumulate neutral lipids. Even combinatorial knockdown of lipins shows mild effects on triacylglycerol accumulation in mature adipocytes.

**Conclusions/interpretation:**

Overall, our data support the hypothesis of alternative pathways for triacylglycerol synthesis in human adipocytes under conditions of repressed lipin expression. We propose that induction of alternative lipid phosphate phosphatases, along with the inhibition of lipid hydrolysis, contributes to the maintenance of triacylglycerol content to near normal levels.

**Electronic supplementary material:**

The online version of this article (doi:10.1007/s00125-016-4018-0) contains peer-reviewed but unedited supplementary material, which is available to authorised users.

## Introduction

Triacylglycerols (TAGs) are neutral lipids that act as the major energy storage molecules, repository for fatty acids, and phospholipid precursors [1]. Adipocytes are the specialised cells for neutral lipid storage and one of their important physiological functions is to buffer the toxicity caused by NEFAs. Excessive calorie intake or genetic disorders can lead to lipid deposition in ectopic tissues, impair their function and lead to dyslipidaemia, insulin resistance and type 2 diabetes [2, 3].

Lipins are Mg^2+^-dependent phosphatidate phosphatases (PAP1) with a central role in lipid metabolism, and catalyse the dephosphorylation of phosphatidate to diacylglycerol (DAG), which can be (1) acylated to form TAG, or (2) used in phospholipids synthesis [4, 5]. A second type of PAP activity is mediated by Mg^2+^-independent transmembrane lipid phosphate phosphatases (LPPs, also known as PAP2), which are thought to regulate signalling properties of phosphatidate and DAG [6].

Fungi, nematodes and insects express one lipin, whereas mammals express three paralogues called lipin-1, -2 and -3 that exhibit distinct but overlapping expression in many mouse and human tissues [5]. Consistent with their key metabolic role, loss of lipin function disrupts TAG production, membrane organisation and phospholipid synthesis in several model organisms [7]. Interestingly, besides their enzymatic functions, lipins also regulate transcription [8–13]. For instance, lipin-1 transcriptional co-regulation of the peroxisome proliferator activated receptor (PPAR)A/PPAR coactivator 1α axis modulates fatty acid oxidation (FAO) in liver [9].

*Lpin1* gene was originally identified as the deficient gene causing lipodystrophy, insulin resistance, peripheral neuropathy and neonatal fatty liver in the *fld* (also known as *Lpin1*) mouse model [14]. Loss of lipin-1 in mice blocks adipogenesis at an early stage preceding TAG accumulation, suggesting a distinct role of lipin-1 in differentiation [15, 16]. Consistently, small interfering RNA (siRNA)-mediated silencing of lipin-1 in mouse 3T3-L1 cells potently inhibits adipogenesis [10, 17]. In contrast, deleterious mutations in the *LPIN1* gene in humans, which lead to recurrent rhabdomyolysis in childhood, do not compromise adipose tissue [18]. The basis of this difference between mice and humans is unknown; it has been hypothesised that it is due to compensation by the other two lipins [18]. Nevertheless, genetic variation in *LPIN1* and *LPIN2*, and reduced *LPIN1* expression levels and PAP1 activity in human adipose tissue have been associated with type 2 diabetes [19–25], which suggests a loss of their protective role against lipotoxicity.

While most of the studies have been performed on mice, very little is known on the roles of lipin-1 in human adipocyte physiology, while there is virtually no information on lipin-2 and -3. This prompted us to investigate the functions of the three lipins in type 2 diabetes and in human adipocytes by studying the effects of loss-of-function.

## Methods

### Reagents

Unless otherwise stated, all reagents were supplied by Sigma-Aldrich Corporation (St. Louis, MO, USA). Human insulin was purchased from NovoNordisk (Bagsværd, Denmark); rosiglitazone from Cayman Chemical (Ann Arbor, MI, USA); and cell culture media from Gibco (Thermo Fisher Scientific, Waltham, MA, USA).

### Human adipose tissue biopsy collection

The cross-sectional study has been previously described [26, 27]. We used a cohort of 71 participants for the gene expression analysis, and of 28 males for protein analysis. They were grouped as: (1) normoweight (BMI between 18.50 and 24.99), (2) obesity (BMI ≥25) and (3) obesity with type 2 diabetes (hereafter type 2 diabetes). Group characteristics are described in electronic supplementary material (ESM) Table 1A and 1B, respectively. Samples were obtained at the Joan XXIII University Hospital (Tarragona, Spain). VAT and SAT samples (gene expression) and SAT samples (protein expression) were obtained during abdominal elective surgical procedures for benign pathologies (cholecystectomy or surgery for abdominal hernia). The Ethics Committee approved the study and informed consent was obtained from all participants. Participants had no systemic disease other than obesity [28] or type 2 diabetes [29]. See ESM Methods for further details.

### Cell culture and differentiation

Simpson–Golabi–Behmel syndrome (SGBS) cells, a well-established system for studies of human adipocyte biology [30, 31], were differentiated as described [30], except that 1 nmol/l of insulin, 0.1 μmol/l cortisol, 2 μmol/l rosiglitazone and 25 nmol/l dexamethasone were used. See ESM Methods for detailed information.

Adipose-derived stem cells were isolated from adipose tissue (*n* = 3 female donors; age (years) 37.4 ± 6.4, BMI (kg/m^2^) 25.9 ± 3.0) from patients undergoing elective liposuction surgery. See ESM Methods for the isolation, proliferation and adipogenic procedure details.

### Quantification of expression levels

Protein content in cell lysates (lysis buffer: 50 mmol/l HEPES pH 7.4, 150 mmol/l NaCl, 4 mmol/l MgCl_2_, 1% Triton X-100 and protease inhibitors) was quantified by using the bicinchoninic acid assay (Pierce, Rockford, IL, USA). See ESM for western blot procedures. Antibodies against lipin family members were previously described [17, 32]. Quantitative PCR was performed as previously described [27], and expressed relative to cyclophilin A and to control. See ESM for detailed information and ESM Table 2A and 2B for commercial reagents.

### Cell Fractionation

For cell fractionation, SGBS cells were grown and differentiated until day 10. Subcellular fractions were obtained by using hypotonic lysis followed by high salt extraction of nuclei. See ESM Methods for further details.

### Gene silencing

Transfections of SGBS cells with siRNA oligonucleotides were carried out by using Lipofectamine RNAiMAX transfection reagent (Thermo Fisher Scientific). Pre-adipocyte knockdowns were performed as a two-shots transfection of a mix of two siRNA duplexes per gene (12.5 nmol/l of each duplex) (see ESM Table 2C): reverse transfection at the start of the experiment and forward transfection the day before confluence. The non-targeting control concentration depended on total siRNA amount of the single and multiple knockdowns.

### Neutral lipid accumulation and metabolism

Data were obtained from differentiated SGBS cells and normalised by protein content. For TAG analysis, cells were processed as previously described [32], except that the supernatant fraction was analysed with the Serum Triglyceride Determination kit (Sigma). Glycerol release to cell culture media was quantified by using the Free Glycerol Determination Kit (Sigma). Fatty acid and glucose incorporation into TAGs, and FAO analysis were performed as previously described [33], with slight modifications (see ESM Methods for details).

### Enzyme assay

Cell lysates (lysis buffer: 50 mmol/l Tris-HCl pH 7.5, 0.25 mol/l sucrose, 10 mmol/l 2-mercaptoethanol, protease inhibitors) were subjected to centrifugation at 1000 *g* for 10 min at 4°C to remove cell debris. Protein concentration was determined by the method of Bradford [34] using BSA as a reference protein. Preparation of the substrate and measure of PAP activity was as previously described [35–37]. See ESM Methods for details. The Mg^2+^-independent LPP activity was measured in the same reaction mixture except that 2 mmol/l EDTA was substituted for 0.5 mmol/l MgCl_2_. The Mg^2+^-dependent PAP1 activity was determined by subtraction of LPPs activity from PAP activity. A unit of PAP activity (expressed as units/mg protein) was defined as the amount of enzyme catalysing the formation of 1 nmol of product/min.

### Metabolomic analysis

SGBS cells were grown, transfected with siRNA as explained above, and differentiated to day 4. In brief, lipids were extracted from lyophilised samples by using dichloromethane/methanol and water. The organic phase (lipidic) was collected, dried under a stream of nitrogen, and resuspended in acetonitrile/isopropanol/water for untargeted LC-MS analysis. Differentially regulated lipids (*p* value <0.05 and fold >2) were retained for compound identification by MS/MS analyses. See ESM Methods for detailed procedures.

### Statistical analysis

Statistical analysis was performed by using the SPSS software version 15 (Chicago, IL, USA). ANOVA, Kruskal–Wallis, Pearson *χ*^2^, Spearman correlation and Linear Stepwise Regression tests were performed for the human cohort analysis, and the General Linear Model Univariate test for in vitro experiments. Statistical power in the cohort analysis was ≥80%. The level of significance was set at *α* = 0.05.

## Results

### Adipose tissue expression levels of the lipin family is altered in type 2 diabetes

**Fig. 1:**
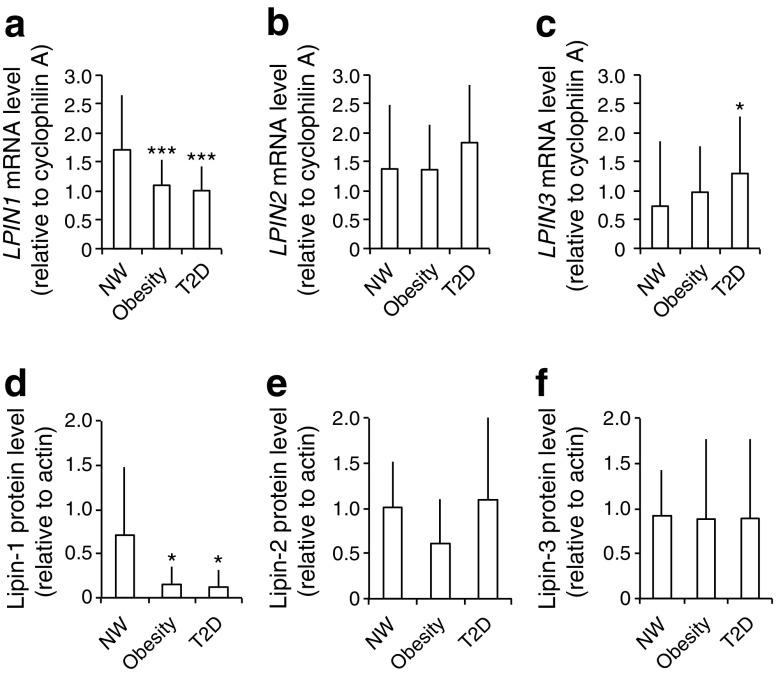
Altered expression of the lipin family in type 2 diabetes adipose tissue. Participants were grouped by BMI and type 2 diabetes (T2D). (**a**–**c**) mRNA expression relative to cyclophilin A and to a calibrator that consisted of a mix of mRNA samples (*n* = 17, 43 and 11 samples for the normoweight [NW], obesity and T2D groups, respectively), and (**d**–**f**) protein expression normalised to actin (*n* = 9, 10 and 9) were quantified in human abdominal subcutaneous adipose tissue. Data represent mean ± SD; *p < 0.05, ***p < 0.001 vs normoweight; ANOVA and Kruskal–Wallis tests

To investigate the roles of lipin paralogues in adipose tissue, we started by examining their gene expression in paired abdominal subcutaneous (SAT) and visceral (VAT) adipose tissue biopsies. As shown in Fig. 1a–c and ESM Fig. 1a–c, *LPIN1* expression was reduced in both SAT (*p* < 0.001) and VAT (*p* = 0.021) in the obesity and type 2 diabetes groups compared with normoweight. *LPIN2* expression was similar among groups. In contrast, SAT *LPIN3* expression was significantly increased in the type 2 diabetes (*p* = 0.018). At the protein level, SAT showed only lipin-1 was downregulated in obesity and in type 2 diabetes compared with normoweight (*p* = 0.034) (Fig. 1d–f).

Correlation analysis showed a negative association of *LPIN1* expression levels with BMI, HOMA-IR and plasma TAG levels. In contrast, expression of *LPIN3* positively correlates with fasting glucose (SAT) and NEFA (VAT) (Table 1). Regression analysis was performed, assessing age and sex as confusing and interacting variables, and showed that (1) SAT *LPIN1* expression depends negatively on HOMA-IR (*R* = 0.466, *p* = 0.005, excluded variables: BMI, TAG, age and sex; *LPIN1*_SAT_ = 0.37 × log_10_HOMA + 0.984), and (2) SAT *LPIN3* expression depends positively on plasma glucose levels (*R* = 0.414, *p* = 0.002, excluded variables: age and sex; $LPIN{3}_{\mathrm{SAT}}=1\times {10}^{\left[1.202\times { \log}_{10}\mathrm{Glucose}-0.961\right]}$).

**Table 1:** Correlation analysis in abdominal SAT and VAT adipose tissue biopsies from 71 participants

Variable	SAT			VAT		
	*LPIN1*	*LPIN2*	*LPIN3*	*LPIN1*	*LPIN2*	*LPIN3*
BMI	−0.349**	−0.021	0.220	−0.283^†^	−0.091	0.034
HOMA-IR	−0.471**	−0.104	−0.006	−0.329*	−0.139	−0.006
Glucose	−0.349**	0.253^†^	0.357**	−0.119	0.189	0.298^†^
Insulin	−0.522***	−0.169	−0.039	−0.340**	−0.179	0.017
Triacylglycerol	−0.319**	−0.018	0.072	−0.202	0.131	0.238
NEFA	−0.095	0.129	0.128	−0.029	0.125	0.336**
Glycerol	−0.188	−0.105	0.144	−0.113	0.097	0.274^†^

Spearman coefficient of the correlation analysis is shown. *p < 0.05, **p < 0.01, ***p < 0.001

Where statistical power is lower than 80%: ^†^ *p* < 0.05

The observed changes of the lipin family expression in type 2 diabetes participants may account for the altered PAP1 activity in adipocytes from these patients. Moreover, *LPIN3* expression is associated with fasting glucose levels. Alterations in lipin-3 protein levels in SAT of participants with type 2 diabetes may be masked by its presence also in the stromal vascular fraction (data not shown).

### The three lipin family members have a role in early human adipogenesis

**Fig. 2:**
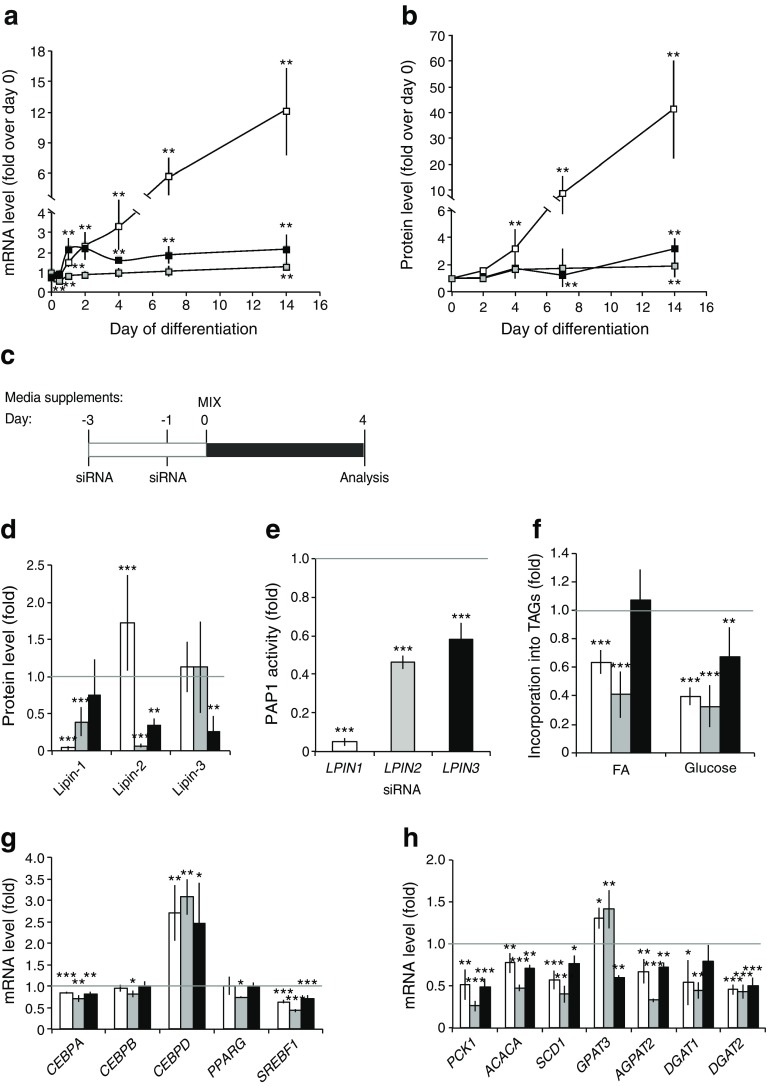
Single knockdowns of lipins in SGBS pre-adipocytes. SGBS pre-adipocytes were induced to differentiate, and mRNA (**a**) and protein (**b**) levels were analysed during adipogenesis at the given time points (*n* = 3). Data represent mean ± SD of fold induction over day 0. (**c**) Knockdowns of single lipin members were performed in pre-adipocytes, adipogenesis was induced (day 0) and cells were collected at day 4. (**d**) Lipin protein levels (*n* = 7), (**e**) PAP1 activity (control: 23.10 ± 5.12 nmol min^−1^ mg^−1^) (*n* = 3), (**f**) fatty acid (FA) and glucose incorporation into TAGs (control, fatty acids: 220.8 ± 185.7 arbitrary units TAG/μg protein, glucose: 3.47 ± 6.30 arbitrary units TAG/μg protein) (*n* = 3), and mRNA levels of (**g**) early adipogenic transcription factors (*n* = 3), and (**h**) lipogenic genes (*n* = 3) were analysed. Gene expression is expressed relative to cyclophilin A and to non-targeting control. Protein expression is normalised to actin levels. Data represent mean ± SD of fold increase over non-targeting controls (set as 1). *p < 0.05, **p < 0.01, ***p < 0.001, General Lineal Model Univariate test. (**a**, **b**) White squares, *LPIN1*; grey squares, *LPIN2*; black squares, *LPIN3*; (**d**–**h**) white bars, *LPIN1* knockdown; grey bars, *LPIN2* knockdown; black bars, *LPIN3* knockdown

Next, we examined the expression of lipins during adipogenesis. Lipin-1 was induced, and lipin-2 and lipin-3 levels were present along SGBS adipogenesis with slight variation (Fig. 2a, b). Thus, SGBS adipocytes, similar to differentiated adipose-derived stem cells (ESM Fig. 1d), express the three lipin paralogues. Finally, under baseline conditions, the three lipins partitioned between the cytosolic, intranuclear and membrane-bound forms in SGBS adipocytes (ESM Fig. 1e).

To address the effects of decreased PAP1 activity on adipogenesis, we depleted each lipin member prior to the induction of adipogenesis in SGBS pre-adipocytes (by using siRNA; see Methods and Fig. 2c). Cells were analysed at day 4 after differentiation to assess adipogenic early events. Protein expression analysis was used to confirm knockdowns and evaluate possible compensatory mechanisms among lipins. Lipin-1 depleted cells responded by compensatory upregulation of lipin-2 protein (Fig. 2d and ESM Fig. 1f), but not *LPIN2* transcript levels (data not shown). Conversely, single lipin-2 and lipin-3 knockdowns led to lower protein levels of the other family members (Fig. 2d). Analysis of PAP1 activity showed that lipin-1 accounted for almost all PAP1 activity (lipin-1-depleted cells showed 5% of PAP1 activity in the control), with lipin-2 and lipin-3 single knockdowns reducing it to 49% and 61%, respectively (Fig. 2e).

Next, we analysed incorporation of fatty acids and glucose into TAGs. While esterification of fatty acids was downregulated in lipin-1- and lipin-2-depleted cells, glucose incorporation into TAGs decreased in all three lipin-depleted cells (Fig. 2f).

Finally, we analysed the expression levels of transcription factors that promote early adipogenesis. *CEBPA* was downregulated in cells depleted of any lipin family member, and *CEBPB* was decreased upon lipin-2 deficiency (Fig. 2g). Under these conditions, gene expression of two key transcription factors regulated by CCAAT/enhancer binding proteins, *PPARG* and *SREBP1* were downregulated (Fig. 2g), and also at the protein level (ESM Fig. 2a, b). *CEBPD* showed a significant upregulation in cells depleted of any lipin family member (Fig. 2g), probably due to the triggering of a compensation mechanism.

Given the role of lipins in neutral lipid biosynthesis, we next explored expression of lipogenic genes. The expression of these genes was significantly downregulated in cells depleted of any lipin family member (Fig. 2h). In contrast, glycerol-3-phosphate acyltransferase was significantly upregulated in the lipin-1 and lipin-2 knockdowns (Fig. 2h). Lipid quantification by mass spectrometry showed that, among the lipid species that were altered, the levels of most DAG (ESM Fig. 2c) and TAG (ESM Fig. 2d) species were downregulated in cells depleted of any lipin compared with controls. Interestingly, phosphatidate levels did not change (data not shown).

Overall, despite the crosstalk between lipin family members, lipin-1 silencing leads to a 95% depletion of PAP1 activity. However, our results point to a role of all three members on early stages of human adipogenesis.

### Single lipin silencing does not block TAG accumulation in fully differentiated adipocytes

**Fig. 3:**
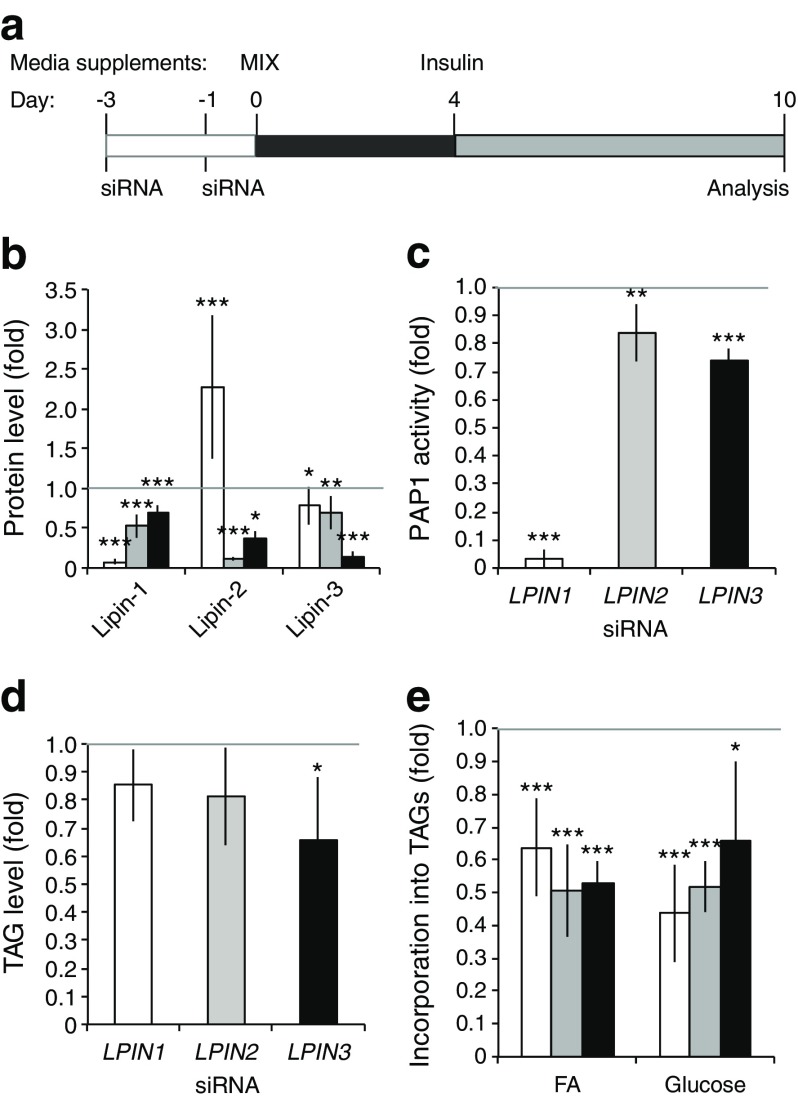
Lipin-depleted SGBS pre-adipocytes can differentiate into adipocytes. (**a**) Knockdowns of single lipin members were performed in pre-adipocytes, adipogenesis was induced (day 0), and cells were collected at day 10. (**b**) Lipin protein levels (*n* = 3), (**c**) PAP1 activity (control: 38.88 ± 1.38 nmol min^−1^ mg^−1^) (*n* = 3), (**d**) total TAG content (control: 110.86 ± 37.29 mmol l^−1^ mg^−1^) (*n* = 3), and (**e**) fatty acid (FA) and glucose incorporation into TAGs (control, fatty acids: 740.6 ± 292.3 arbitrary units TAG/μg protein, glucose: 3.73 ± 2.60 arbitrary units TAG/μg protein) (*n* = 3) were analysed. Data represent mean ± SD of fold increase over non-targeting controls (set as 1); *p < 0.05, **p < 0.01, ***p < 0.001; General Lineal Model Univariate test. White bars, *LPIN1* knockdown; grey bars, *LPIN2* knockdown; black bars, *LPIN3* knockdown

Next, we analysed the effects of lipin silencing in SGBS pre-adipocytes on late adipogenesis. Similarly to the above experiments, we depleted each lipin member prior to induction of adipogenesis in SGBS pre-adipocytes, and cells were analysed at day 10 after differentiation (Fig. 3a), where protein downregulation of lipins still persisted (Fig. 3b and ESM Fig. 3a).

At day 10, lipin-1 depleted cells still responded by compensatory upregulation of lipin-2 protein (Fig. 3b and ESM Fig. 3a). Additionally, both single lipin-2 and lipin-3 knockdowns led to lower protein levels of the other lipins. PAP1 activity analysis showed that lipin-1 accounted for almost all PAP1 activity (lipin-1-depleted cells showed 3% of the activity in the control). Lipin-2 and lipin-3 single knockdowns reduced PAP1 activity only slightly (84% and 74%, respectively) (Fig. 3c).

Next, we assessed neutral lipid accumulation. Depletion of any lipin triggered a slight reduction (15% to 34%) in TAG accumulation (Fig. 3d). Expression of key transcription factors for lipogenesis (ESM Fig. 3a, b) and genes of lipid biosynthesis (ESM Fig. 3c) were altered to a lesser extent compared with the effects observed at day 4.

Finally, fatty acid esterification into TAGs and α-glycerophosphate synthesis/de novo lipogenesis (DNL) were downregulated in cells depleted of any lipin (Fig. 3e). Compared with day 4, lipin-3 depletion also showed reduced fatty acid esterification into TAGs at day 10.

Overall, the effects of lipin knockdown were mild on fully differentiated adipocytes suggesting that cells overcame the initial impairment of lipid gene expression. This may be due to the existence of enough remnant protein amounts of lipins, although PAP1 activity at day 10 was still almost absent in the lipin-1 knockdowns.

### Combinatorial silencing of lipins does not further alter the phenotype of fully differentiated adipocytes

We then asked whether compensation by other lipin family members might help cells to recover the initial alterations shown at day 4 after adipogenesis. We used a combinatorial knockdown to deplete combinations of two of the three lipins in SGBS pre-adipocytes (see Methods), and analysed cells at day 10 of differentiation (as in Fig. 3a).

**Fig. 4:**
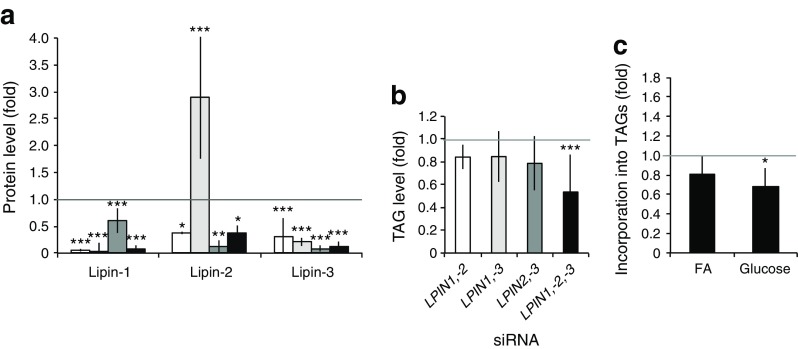
Combinatorial depletion of lipins in SGBS pre-adipocytes after full differentiation. Multiple knockdowns of lipin members were performed in pre-adipocytes, adipogenesis was induced (day 0) and cells were collected at day 10. (**a**) Lipin protein levels (*n* = 3–6), (**b**) total TAG content (controls: 71.11 ± 36.40 and 101.79 ± 76.15 mmol l^−1^ mg^−1^ for double and triple knockdowns, respectively) (*n* = 5), and (**c**) fatty acid (FA) and glucose incorporation into TAGs in the triple knockdown (control, fatty acids: 493.4 ± 226.4 arbitrary units TAG/μg protein, glucose: 3.51 ± 2.26 arbitrary units TAG/μg protein) (*n* = 3) were analysed. Data represent mean ± SD of fold increase over non-targeting controls (set as 1); *p < 0.05, **p < 0.01, ***p < 0.001; General Lineal Model Univariate test. White bars, *LPIN1* and *LPIN2* knockdown; light grey bars, *LPIN1* and *LPIN3* knockdown; dark grey bars, *LPIN2* and *LPIN3* knockdown; black bars, triple knockdown

Double lipin-1 and lipin-3 knockdowns led to an upregulation of lipin-2 protein levels (Fig. 4a and ESM Fig. 4). This compensatory pattern hindered the efficiency of lipin-2 knockdown in the double (lipin-1 and -2) and triple knockdown cells. Nevertheless, PAP1 activity in the triple knockdown was residual compared with the control (0.18 ± 0.31 vs 39.29 ± 3.42 nmol min^−1^ mg^−1^).

Despite the almost complete lack of PAP1 activity, neutral lipid accumulation was not totally compromised in the triple knockdown (Fig. 4b). This is in agreement with the fact that deleterious mutations in *LPIN1* do not affect adipose tissue development in humans, and it suggests that it is unlikely to be due to a compensatory upregulation of lipin-2 (Fig. 2d), since the double lipin-1 and -2 knockdown shows a similar reduction in the content of neutral lipids (Figs 3d, 4b).

Moreover, depletion of the three lipins did not significantly affect fatty acid esterification into TAGs and only slightly downregulated α-glycerophosphate synthesis/DNL (Fig. 4c), pointing to an improvement compared with day 4 (day 4 incorporation of fatty acids: 0.52 ± 0.35, *p* = 0.006, and of glucose: 0.32 ± 0.15, *p* < 0.001, fold over the control level).

### Consumption of TAGs is reduced in lipin-depleted SGBS adipocytes

**Fig. 5:**
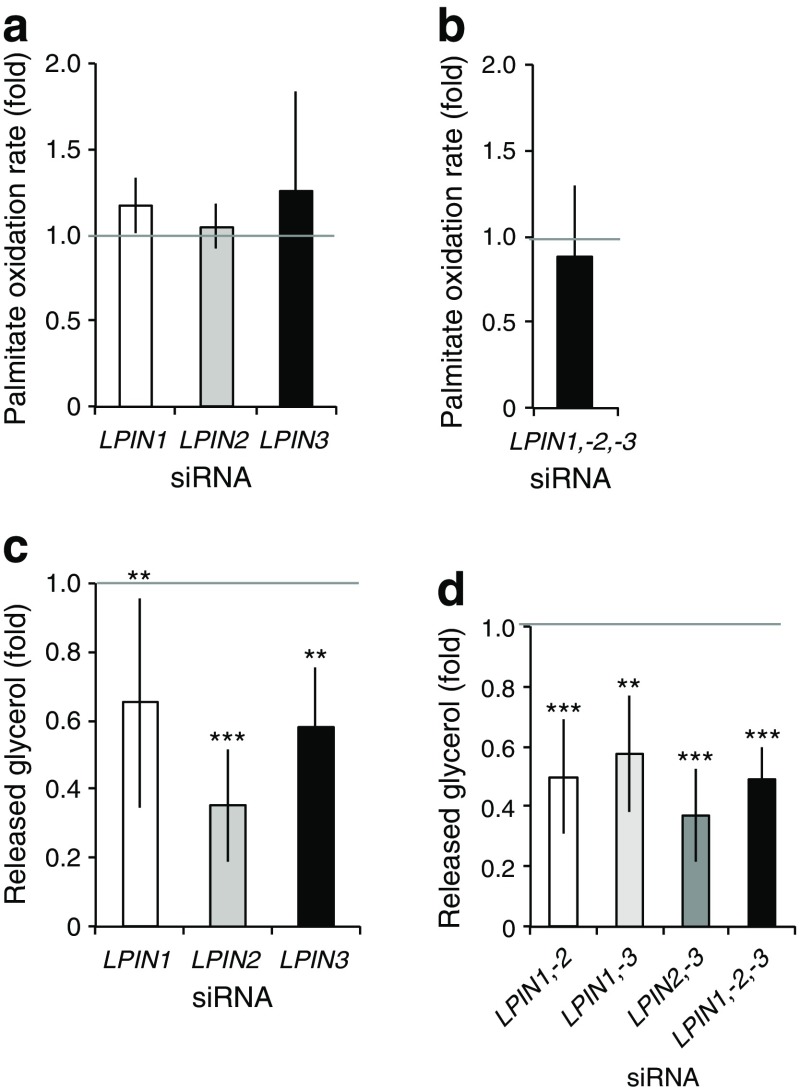
Consumption of neutral lipids in fully differentiated lipin-depleted SGBS pre-adipocytes. Knockdown of single, double and triple lipin family members was performed in pre-adipocytes, adipogenesis was induced (day 0) and cells were collected at day 10. Palmitate oxidation rates in (**a**) single (control: 0.77 ± 0.37 nmol h^−1^ mg^−1^) (*n* = 3), and (**b**) triple knockdowns (control: 0.63 ± 0.28 nmol h^−1^ mg^−1^) (*n* = 3); glycerol release in (**c**) single (control: 0.42 ± 0.12 mmol l^−1^ mg^−1^) (*n* = 3), and (**d**) combinatorial knockdowns (controls, double: 0.36 ± 0.21, and triple: 0.21 ± 0.07 mmol l^−1^ mg^−1^) (*n* = 5) were analysed. Data represent mean ± SD of fold increase over non-targeting controls (set as 1); **p < 0.01, ***p < 0.001; General Lineal Model Univariate test. (**a**, **c**) White bars, *LPIN1* knockdown; grey bars, *LPIN2* knockdown; black bars, *LPIN3* knockdown; (**b**, **d**) white bars, *LPIN1* and *LPIN2* double knockdown; light grey bars, *LPIN1* and *LPIN3* double knockdown; dark grey bars, *LPIN2* and *LPIN3* double knockdown; black bars, triple knockdown

Lipid droplets accumulate neutral lipids in a dynamic manner, with a balance among lipid biosynthesis, lipid hydrolysis and FAO in mitochondria. To test whether cells protect intracellular lipids by reducing its consumption, we analysed the rates of palmitate oxidation. The results show similar FAO rates in the control, single (Fig. 5a) and triple knockdown (Fig. 5b). In contrast, levels of glycerol release were downregulated compared with controls in the single (Fig. 5c), and in all combinatorial knockdowns (Fig. 5d). Thus, reduced consumption may contribute to the accumulation of neutral lipids in lipin-depleted adipocytes.

### The LPP family is upregulated under conditions of repressed lipin expression

**Fig. 6:**
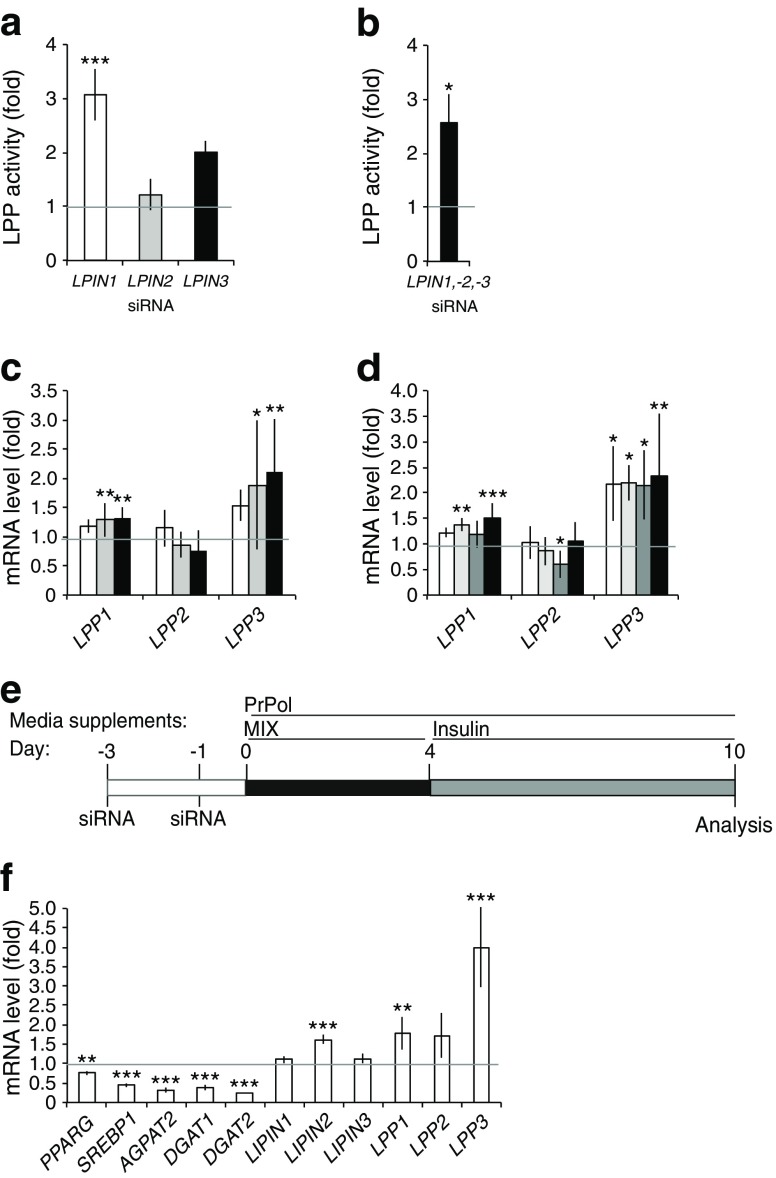
Induction of the LPP family under conditions of repressed lipin expression. Knockdowns of single, double and triple lipin family members were performed in pre-adipocytes, adipogenesis was induced (day 0) and cells were collected at day 10. LPP activity in (**a**) the single (control: 6.01 ± 0.60 nmol min^−1^ mg^−1^) (*n* = 3) and (**b**) triple knockdowns (control: 4.82 ± 0.53 nmol min^−1^ mg^−1^) (*n* = 3), and expression levels of the LPP family in (**c**) the single (*n* = 7) and (**d**) combinatorial knockdowns (*n* = 4–7) were analysed. (**e**) SGBS pre-adipocytes were induced to differentiate in presence of 100 μmol/l of propranolol (PrPol). (**f**) mRNA levels of lipogenic genes were analysed (*n* = 3). Data represent mean ± SD of fold increase over non-targeting controls (**a**–**d**) or non-treated control (**f**); *p < 0.05, **p < 0.01, ***p < 0.001; General Lineal Model Univariate test. (**a**, **c**) White bars, *LPIN1* knockdown; grey bars, *LPIN2* knockdown; black bars, *LPIN3* knockdown; (**b**, **d**) white bars, *LPIN1* and *LPIN2* double knockdown; light grey bars, *LPIN1* and *LPIN3* double knockdown; dark grey bars, *LPIN2* and *LPIN3* double knockdown; black bars, triple knockdown; (**f**) white bars, propranolol-treated cells

Next, we tested whether the Mg^2+^-independent LPPs could provide an alternative pathway for DAG synthesis. LPP activity was highly induced in the lipin-1 single knockdown (3.50 ± 1.18-fold increase over control levels) (Fig. 6a), and in the triple knockdown (2.74 ± 0.97-fold increase) at day 10 of differentiation (Fig. 6b).

Moreover, the expression of *LPP3* (also known as *Plpp3/PPAP2B*) was upregulated in the single (Fig. 6c) and in the combinatorial lipin knockdowns at day 10 (Fig. 6d) and, similarly to lipogenic genes, upregulation was higher at day four (ESM Fig. 5a). This was confirmed by using other commercial sources of lipin-1 siRNA (ESM Fig. 5b) and analysing LPP expression at day four of differentiation (ESM Fig. 5c).

Propranolol, a nonspecific ß-blocker, is an effective PAP1 activity inhibitor and modestly effective as LPP inhibitor, with the LPP3 isoform being more sensitive to inhibition [36]. Propranolol treatment during adipogenesis (Fig. 6e) altered lipogenic gene expression (Fig. 6f) and blocked lipid droplet formation in SGBS adipocytes (ESM Fig. 5d).

## Discussion

Herein we have addressed the contribution of the lipin paralogues to human adipogenesis. Decreased lipin expression levels and PAP1 activity in adipose tissue have been linked with insulin resistance [23–25]. In our cohort, adipose tissue expression confirmed that lipin-1 is altered in type 2 diabetes and that *LPIN1* is negatively associated with insulin resistance. Moreover, we show that *LPIN3* transcript levels are positively related with fasting glucose levels. This prompted us to analyse separately the effects of gene silencing of each lipin paralogue in adipogenesis.

Previous studies have established that lipin-1 plays a major role in fat metabolism in rodents, with lipin-1 deficiency causing lipodystrophy features in mice and rats [14, 38]. The role of lipins in human adipogenesis is still undefined: mutations in the *LPIN1* gene have not yet been detected in human lipodystrophy [39, 40]. Moreover, deleterious *LPIN1* mutations cause paediatric rhabdomyolysis while fat distribution, average weight and plasma biochemical variables are normal [18]. Our data suggest that the lack of an essential role for PAP1 activity in human adipogenesis is not due to compensation by lipin-2 or lipin-3. We find that the triple lipin silencing in SGBS pre-adipocytes maintains the ability to incorporate fatty acids into TAG and accumulate neutral lipids despite loss of nearly all PAP1 activity.

SGBS pre-adipocytes are differentiated in the absence of serum and, therefore, neutral lipids should be obtained from DNL. Besides, although glyceroneogenesis is the major pathway for glycerol synthesis in the mature adipocyte [41], during adipogenesis glycolysis may account for an important source of α-glycerophosphate. Thus, glucose incorporation into TAGs may account for DNL, but also for α-glycerophosphate synthesis via glyceroneogenesis and glycolysis. Downregulated α-glycerophosphate synthesis/DNL points to broader effects of lipin silencing other than TAG synthesis. Moreover, the putative intranuclear roles of lipin-1, -2 and -3 in SGBS adipocytes add some complexity to the above.

The lipin family members cooperate for optimal PAP activity in mouse adipose tissue [42], liver [43, 44], brain [43] and human primary myoblasts [45]. Our data show that human lipin-1 accounts for most PAP1 activity in SGBS adipocytes and that upregulation of lipin-2 in lipin-1-depleted cells does not compensate for PAP1 activity. By contrast, downregulation of PAP1 activity in lipin-2 and lipin-3 single knockdowns cannot be fully attributed to their own depletion since they also downregulate lipin-1.

Activation of non-lipin compensatory pathways may also mask the lipin phenotypes. Nevertheless, (1) we found no evidence, at the mRNA level, of upregulation of the monoacylglycerol *O*-acyltransferase pathway that can generate DAG from monoacylglycerol [46, 47] (data not shown); and (2) cholesteryl esters levels are likely not upregulated since the effect of lipin knockdown on neutral lipid content looks similar by Oil Red O staining (ORO) and TAG content (ORO: 0.76 ± 0.06, and TAG: 0.85 ± 0.13 in the lipin-1 knockdown, and ORO: 0.74 ± 0.12, and TAG: 0.54 ± 0.33 in the triple lipin knockdown, compared with the control set as 1). In contrast, LPP activity was induced in the lipin knockdowns, possibly due to an earlier upregulation of their transcript levels (observed by day 4). Moreover, inhibition of both lipins and LPPs with propranolol completely blocked lipid droplet formation, although this fact must be taken with caution because of the non-specificity of this compound. LPPs hydrolyse phosphatidate as well as different substrates, and act on the outer surface of plasma membrane and in the luminal surface of endoplasmic reticulum (ER) and Golgi membranes [6] and thus, it is not clear if they can have access to the phosphatidate formed from glycerol phosphate and acyl-CoA. However, in yeast, TAG synthesised both in the cytosolic and luminal leaflets of the ER membranes are efficiently packed into lipid droplets [48].

Another strategy to protect the lipid storage when glycerolipid synthesis is compromised might be downregulation of lipid hydrolysis and FAO. Lipin-1 regulates basal lipolysis [49], and modulates FAO transcript expression levels [9]. We show that all combinatorial knockdowns of lipins downregulate basal lipolysis to a greater extent than reduction of neutral lipid content.

In summary, we confirm that adipose tissue expression of the lipin family is altered in type 2 diabetes. Furthermore, loss of nearly all PAP1 activity, due to combined lipin-1, -2 and -3 knockdown, has only mild effects on final adipogenesis and lipid accumulation in SGBS cells. Conversely, lipin-2 and lipin-3 may contribute little to total PAP activity, but still play a role in early adipogenesis. Our results suggest a compensation strategy to accumulate near normal neutral lipid levels, activating other pathways (such as LPPs) and inhibiting TAG hydrolysis. More work is clearly required to decipher whether LPPs or other unknown pathways may compensate lack of PAP1 activity.

## Electronic supplementary material

Below is the link to the electronic supplementary material.

ESM Methods (PDF 157 kb)

ESM Table 1 (PDF 89.8 kb)

ESM Table 2 (PDF 64.4 kb)

ESM Fig. 1 (PDF 498 kb)

ESM Fig. 2 (PDF 318 kb)

ESM Fig. 3 (PDF 365 kb)

ESM Fig. 4 (PDF 186 kb)

ESM Fig. 5 (PDF 2.26 mb)

## Abbreviations

DAG: Diacylglycerol
DNL: De novo lipogenesis
ER: Endoplasmic reticulum
FAO: Fatty acid oxidation
LPP: Lipid phosphate phosphatases
ORO: Oil Red O staining
PAP: Phosphatidate phosphatase
PPAR: Peroxisome proliferator activated receptor
SGBS: Simpson–Golabi–Behmel syndrome
siRNA: Small interfering RNA
SAT: Subcutaneous adipose tissue
TAG: Triacylglycerol
VAT: Visceral adipose tissue
