# Supplementary material for: Redundant roles of the phosphatidate phosphatase family in triacylglycerol synthesis in human adipocytes
Source: Diabetologia. 2016 Jun 25;59:1985–94. doi: 10.1007/s00125-016-4018-0 (PMC4969345; doi:10.1007/s00125-016-4018-0)
Supplement: Supplementary file 1 — (PDF 157 kb) [file 125_2016_4018_MOESM1_ESM.pdf]

**ESM Methods**

## Human adipose tissue biopy collection

*Selection of patients*— For the gene expression study, a group of 71 participants was recruited at Joan XXIII University Hospital (Tarragona, Spain) and Sant Pau i Santa Tecla Hospital (Tarragona, Spain). All participants were of Caucasian origin and reported that their body weight had been stable for at least 3 months before the study. They had no systemic disease other than obesity or type 2 diabetes, and all had been free of any infections in the month before the study. Liver and renal diseases were specifically excluded by biochemical workup.

The study included 17 normoweight, 43 individuals with obesity, and 11 individuals with type 2 diabetes, matched for age and gender (ESM Table 1A). Participants were classified by BMI according to the World Health Organization criteria [1]. Eleven patients were classified as having type 2 diabetes according to the American Diabetes Association criteria [2]. Variability in metabolic control was assessed by stable glycated haemoglobin A1c values during the previous 6 months. No patients were being treated with thiazolidinedione. Pharmacological treatment of the patients with type 2 diabetes was as follows: insulin, 9.1%; oral hypoglycaemic agents, 54.5%; statins, 63.6%; blood pressure- lowering agents, 54.5%.

Sample size was calculated, based in a previous study [3], to achieve a difference in normalized gene expression levels between the studied groups of 0.3 or greater with a confidence level of 95% and a statistical power of 80%.

Age and sex were assessed as interaction and confusing variables in the regression analysis. The global signification test was used to analyse interactions and for proposing the reference model. Residual analysis was performed to evaluate the models and data exclusion. The final regression model was chosen by comparing reduced models with the reference model.

For the protein expression levels of the lipin family, a group of 28 male participants was recruited at Joan XXIII University Hospital (Tarragona, Spain). The study included 9 normoweight, 10 individuals with obesity, and 9 individuals with type 2 diabetes, matched for age (ESM Table 1B).

*Anthropometric measurements*— Height was measured to the nearest 0.5 cm and body weight to the nearest 0.1 kg. BMI was calculated as weight (kilograms) divided by height (meters) squared. Waist circumference was measured midway between the lowest rib margin and the iliac crest.

*Collection and processing of samples*— VAT (omental) and SAT (anterior abdominal wall) samples were obtained from all individuals for the gene expression analysis, and SAT samples for the protein expression analysis. Samples were obtained during abdominal elective surgical procedures (cholecystectomy or surgery for abdominal hernia). All patients had fasted overnight. Blood samples were collected before the surgical procedure from the antecubital vein: 20 ml of blood with EDTA (1 mg/ml), and 10 ml of blood in silicone tubes. Fifteen millilitres of collected blood were used for the separation of plasma. Plasma and serum samples were stored at -80 C until analytical measurements were performed. Five millilitres of blood with EDTA were used to determine glycated haemoglobin A1c. Adipose tissue samples were collected, washed in 1X PBS, immediately frozen in liquid nitrogen, and stored at -80 C.

*Analytical methods*— Plasma glucose, cholesterol, and triacylglycerol levels were determined in an autoanalyzer (Hitachi 737; Roche Molecular Bio- chemicals, Marburg, Germany) using the standard enzyme methods. Non-esterified free fat acid (NEFA) serum levels were determined in an autoanalyzer (Advia 1200; Siemens AG, Munich, Germany) using an enzymatic method developed by Wako Chemicals (Neuss, Germany). Plasma glycerol levels were analyzed by using a free glycerol determination kit, a quantitative enzymatic determination assay (Sigma-Aldrich Corp., St. Louis, MO). Intra- and interassay coefficients of variation were less than 6% and less than 9.1%, respectively. Plasma insulin was determined by RIA (Coat-A-Count insulin; Diagnostic Products Corp., Los Angeles, CA) in all participants, except in insulin-treated type 2 diabetes patients. The homeostasis model assessment of insulin resistance (HOMA-IR) was determined as [glucose (mmol/l) x insulin (mIU/l)]/22.5 [4].

## Cell culture and differentiation

*Differentiation of SGBS cells*— Preadipocytes were grown in serum-containing medium (DMEM/F12 supplemented with 10% foetal bovine serum, 33 μmol/l biotin, 17 μmol/l pantothenate and antibiotics) in a humidified 37 °C incubator with 5% $CO_2$, until reaching confluence. Cells were seeded at 25,000 cells per $cm^2$ and reached confluence in three days. To induce adipose differentiation cells were repeatedly washed with PBS buffer and cultured for four days in serum-free, basal medium supplemented with 33 μmol/l biotin, 17 μmol/l pantothenate, 1 nmol/l insulin, 200 pmol/l triiodothyronine, 0.1 μmol/l cortisol, 0.01 mg/ml transferrin, 0.5 mmol/l IBMX, 2 μmol/l rosiglitazone and 25 nmol/l dexamethasone (differentiation medium). Cells were cultured in adipocyte maintenance medium for one week (differentiation medium without IBMX, dexamethasone and rosiglitazone). The medium was changed twice a week.

*Human adipose stem cell isolation*—All tissues were obtained from patients undergoing elective liposuction surgery. Adipose-derived stem cells (ASC) were isolated from adipose tissue (n=3), according to published protocols [5]. Briefly, ASC were obtained from washed lipoaspirate tissues by collagenase digestion, from the Biobank of the Joan XXIII University Hospital. The resulting stromal vascular cells were cultured in medium consisting of Dulbecco's modified Eagle's medium (DMEM)/F12, 10% fetal bovine serum (FBS), plus antibiotics at 37 °C, 5% $CO_2$ overnight. The following day, the ASC were rinsed with warm PBS and maintained until 80–90% confluent. The ASC cultures were collected by trypsin digestion and aliquots of $10^6$ cells cryopreserved in liquid nitrogen until required for experimentation.
Thawed ASC cells were transferred to culture vessels at a density of $5x10^3$ cells/$cm^2$ in preadipocyte medium (low glucose DMEM supplemented with 10% FBS, 15 mmol/l HEPES, 2.5 ng/ml Fibroblast growth factor-2, 33 μmol/l biotin, 17 μmol/l pantothenate and antibiotics). The cells were maintained in a humidified tissue culture incubator at 37 °C with 5% $CO_2$. The medium was replaced every second day until the cells reached 80% confluence. Cells were collected by trypsin digestion for inducing adipogenic differentiation.

*Differentiation of ASCs*— The ASCs were used at passage 5. To induce adipogenesis, confluent cultures of ASCs were cultured for 2 days in adipocyte differentiation medium (DMEM/F-12 supplemented with 10% FBS, 5 μmol/l rosiglitazone, 1 nmol/l dexamethasone, 0.5 mmol/l 1-methyl-3-isobutylxanthine (IBMX), 0.01 mg/ml transferrin, 1 nmol/l human insulin, 33 μmol/l biotin, 17 μmol/l pantothenate and antibiotics). The induced cells were re-fed every 3 days with adipocyte maintenance

medium (adipocyte differentiation medium without IBMX, and dexamethasone), during 12 days.

## Quantification of expression levels

*Western blot analysis*— 10 μg of total protein per lane were resolved by electrophoresis in 8 to 12% SDS–PAGE, transferred to nitrocellulose membranes (Whatman), blocked in either 5% non-fat dry milk in PBS containing 0.01% Tween 20 or following manufacturer´s recommendations for 1 h at room temperature, incubated with the primary antibody overnight at 4 °C and secondary for 1 h. The western blots were developed with Super-Signal West Femto Maximum Sensitivity Substrate (Pierce). Images were quantified by using ImageJ v.1.48 (Wayne Rasband, National Institutes of Health, USA). Actin and GAPDH protein levels were used for normalisation.

*qPCR*—Total RNA was extracted by using an RNeasy Lipid Tissue Midi Kit (total adipose tissue) and RNeasy Tissue Mini Kit (SGBS cells) (QIAGEN, Germantown, MD, USA). Total RNA was transcribed to cDNA by using a High-Capacity cDNA Reverse Transcription Kit (Applied Biosystems, LifeTechnologies Corporation). Real time qPCR analysis was performed with duplicates on a 7900HT Fast Real-Time PCR System using hydrolysis probes (Applied Biosystems, ESM Table 2C). SDS software 2.3 and RQ Manager 1.2 (Applied Biosystems) were used to analyse the results with the comparative quantification cycle (Cq) method ($2^{-\Delta\Delta Cq}$), and cyclophilin A as reference gene. For the human cohort analysis, samples from different groups were randomised at every qPCR run.

## Cell Fractionation

For cell fractionation, cells were processed as described previously [6], with some modifications. The whole process was performed in ice and centrifugations were performed at 16,000 x g and 4˚C. Mainly, SGBS cells were grown and differentiated in two wells from 6-well plates, washed with PBS, scrapped in 75 μl of 10 mmol/l Tris-HCl (pH 7.4), 0.2 mmol/l AEBSF, 1.5 mmol/l $MgCl_2$, 10 mmol/l NaCl, 0.5 mmol/l dithiothreitol (DTT). Lysed cells were incubated in ice for 15 minutes vortex mixed, and centrifuged 10 seconds. The supernatant was kept as cytosolic fraction and the pellet was resuspended in 20 mmol/l Tris-HCl (pH 7.4), 25% glycerol, 0.2 mmol/l AEBSF, 1.5 mmol/l $MgCl_2$, 420 mmol/l NaCl, 0.5 mmol/l DTT, 0.2 mmol/l EDTA. The lysate was incubated in ice for 20 minutes and centrifuged 2 minutes. The supernatant was kept as nuclear fraction and the pellet was resuspended in $H_2O$ as membrane bound proteins.

## Neutral lipid accumulation and metabolism

Fatty acid and glucose incorporation into TAGs was measured by incubating adipocytes for 16 h at 37°C in serum-free medium containing 0.5 mmol/l palmitate and 1 μCi/ml [1-14C] palmitate bound to 1% bovine serum albumin (BSA) or 5 mmol/l glucose and 1 μCi/ml [1-14C] glucose. Cells were then washed in PBS and lipids were extracted and separated by TLC to measure the incorporation of labelled fatty acid into TAGs, as described [7]. Data are expressed as arbitrary units normalised by protein content. For FAO analysis, adipocytes were washed in KRBH 0.1% BSA, preincubated at 37 ºC for 30 min in KRBH 1% BSA, and washed again in KRBH 0.1% BSA. Cells were then incubated for 4 h at 37 ºC with fresh KRBH containing 5 mmol/l glucose and 0.5 mmol/l carnitine plus 0.25 mmol/l palmitate and 1 μCi/ml [1-14C]palmitate bound to

1% BSA. Palmitate oxidation to $CO_2$ measurements were performed as previously described [7], and expressed as nmol of $CO_2$ $h^{-1}$ $mg^{-1}$ of protein.

## Enzyme assay

The radioactive substrate [32P]phosphatidate was enzymatically synthesized from 1,2-dioleoyl-sn-glycerol and [γ-32P]ATP with Escherichia coli diacylglycerol kinase. PAP activity was measured with cell extracts at 37 C for 20 min in a total volume of 100 µl containing 50 mmol/l Tris-HCl (pH 7.5), 0.5 mmol/l MgCl2, 10 mmol/l 2-mercaptoethanol, 0.2 mmol/l [32P]phosphatidate (5,000 cpm/nmol), and 2 mmol/l Triton X-100.

## Metabolomic analysis

SGBS cells were grown in 6-well plates, transfected with siRNA as explained above, and differentiated to day 4. Three wells from a 6-well plate were used per condition, and cells were washed with 1X phosphate buffer saline and collected by scrapping with 355 µl of methanol. Collected cells were sonicated, and an aliquot of 40 µl was used for protein quantification. The remaining volume was frozen and kept at -80 C until the analysis. Four replicas were performed.

*Lipid extraction method*— Lipids were extracted from lyophilized samples by adding 570 µl of a cold mixture of dichloromethane/methanol (2:1 v/v). The resulting suspension was vortexed and bath-sonicated for 5 min. We subsequently added 120 µl of cold water, vortex samples again and organic and aqueous layers were allowed to equilibrate for 10 min at room temperature. Cell lysates were centrifuged (15,000 rpm, 15 min at 4 C), and the organic phase (lipidic) was collected for drying under a stream of nitrogen. Lipid pellets were resuspended in 300 µl of acetonitrile/isopropanol/water (65:30:5 v/v) for LC-MS analysis. 100 µl of culture media was lyophilized and subsequently resuspended in dichloromethane/methanol (2:1 v/v) following the same procedure as that used for cells, with the exception that lipid pellets were resuspended in 200 µl (acetonitrile: isopropanol: water (65:30:5 v/v).

*LC/MS analysis*— Untargeted LC/MS analyses were performed using an UHPLC system (1200 series, Agilent Technologies) coupled to a 6550 ESI-QTOF MS (Agilent Technologies) operating in positive (ESI+) or negative (ESI–) electrospray ionization mode. Lipids were separated by reverse phase chromatography with an Acquity UPLC C8 column (150 x 2.1 mm, 1.8 µm). Mobile phase A = water/acetonitrile (60:40) (10 mmol/l ammonium formate and 0.1% formic acid) and B = isopropanol/acetonitrile (95:5) (10 mmol/l ammonium formate, 0.1% formic acid and 0.1% H2O). Solvent modifiers, such as 0.1% formic acid and 10 mmol/l ammonium formate, were used to enhance ionization, as well as to improve the LC resolution in both positive and negative ionization modes. The elution gradient started at 32% B (time 0–1 min), increased to 60% of B (time 1–4 min) and increased again to 100% B over 11 min (time 4–15 min). The injection volume was 2 µl for cell lipids and 5 µl for media lipids. ESI conditions: gas temperature, 150 C; drying gas, 13 l/min; nebulizer, 35 psig; fragmentor, 150 V; and skimmer, 65 V. The instrument was set to work over the m/z range 50–1200 with an acquisition rate of 3 spectra/sec. For compound identification, MS/MS analyses were performed in targeted mode, and the instrument set to acquire spectra over the m/z range 50–1000, with a default iso width (the width at half-maximum of the quadrupole mass bandpass used during MS/MS precursor isolation) of 4 m/z. The collision energy was fixed at 20 V.

*Lipidomic data analysis*— LC/MS (ESI+ and ESI− mode) data were processed using the XCMS [8] software to detect and align mzRT features. A feature is defined as a molecular entity with a unique m/z and a specific retention time. XCMS analysis of these data provided a matrix containing the retention time, m/z value, and integrated peak area of each feature for each sample of cells and culture medium. We constrained the initial number of features by means of the following criteria: only features above an intensity threshold of 3,400 counts were retained for further statistical analysis. Quality control samples (QCs) consisting of pooled cells from each condition were injected at the beginning and periodically every four samples. The performance of the LC/MS platform for each mzRT feature detected in the cell culture samples was assessed by calculating the relative standard deviation of these features on pooled samples (CVQC), following Vinaixa et al. [9]. Next, the intensities of the mzRT features were compared using a One-way ANOVA for each differentiation day separately, correcting for multiple testing using Tukey's 'Honest Significant Difference' method. Differentially regulated lipids (p value<0.05 and fold>2) were retained for further tandem MS characterization. Lipid structures were identified by matching tandem MS spectra against reference standards in HMDB [10], LIPIDMAPS [11] or LipidBlast [12] databases, or using CFM-ID software [13].

**Statistical analysis**

ANOVA, Kruskal–Wallis, and Pearson $\chi 2$ tests were performed for the human cohort analysis, and the General Linear Model Univariate test and Student T-test for in vitro experiments. Statistical power in the cohort analysis was ≥80%. The level of significance was set at $\alpha$=0.05.

**Abbreviations**

(AEBSF) 4-(2-aminoethyl) benzenesulfonyl fluoride, (ASC) adipose-derived stem cells, (BSA) bovine serum albumin, (DTT) dithiothreitol, 1-methyl-3-isobutylxanthine (IBMX),

**Bibliography**


1. World Health Organization (2000) Obesity: preventing and managing the global epidemic. Report of a WHO Consultation Geneva. WHO Tech Rep Ser 894. 1st ed. Geneva: World Health Organization.

2. International Diabetes Federation (2006) IDF Consensus Worldwide Definition of the Metabolic Syndrome: https://www.idf.org/webdata/docs/MetS_def_update2006.pdf, accessed 24 November 2015.

3. Miranda M, Chacón MR, Gómez J, et al. Human subcutaneous adipose tissue LPIN1 expression in obesity, type 2 diabetes mellitus, and human immunodeficiency virus--associated lipodystrophy syndrome. Metabolism. 2007 Nov;56:1518-26.

4. Matthews DR, Hosker JP, Rudenski AS, Naylor BA, Treacher DF, Turner RC (1985) Homeostasis model assessment: insulin resistance and ß-cell function from fasting plasma glucose and insulin concentrations in man. Diabetologia 28:412–419

5. Pachón-Peña G, Yu G, Tucker A, et al (2011) Stromal stem cells from adipose tissue and bone marrow of age-matched female donors display distinct immunophenotypic profiles. J Cell Physiol 226:843-851

6. Andrews NC, Faller DV (1991) A rapid micropreparation technique for extraction of DNA-binding proteins from limiting numbers of mammalian cells. Nucleic Acids Res 19:2499.

7. Sebastián D, Guitart M, García-Martínez C, et al. (2009) Novel role of FATP1 in mitochondrial fatty acid oxidation in skeletal muscle cells. J Lipid Res 50:1789-1799

8. Smith CA, Want EJ, O'Maille G, Abagyan R, Siuzdak G (2006) XCMS: processing mass spectrometry data for metabolite profiling using nonlinear peak alignment, matching, and identification. Anal Chem 78:779-787

9. Vinaixa M, Samino S, Saez I, Duran J, Guinovart JJ, Yanes O (2012) A Guideline to Univariate Statistical Analysis for LC/MS-Based Untargeted Metabolomics-Derived Data. Metabolites 2:775-795

10. Wishart DS, Tzur D, Knox C, et al. (2007) HMDB: the Human Metabolome Database. Nucleic Acids Res 35:D521-526

11. Fahy E, Sud M, Cotter D, Subramaniam S (2007) LIPID MAPS online tools for lipid research. Nucleic Acids Res 35:W606-612

12. Kind T, Liu KH, Lee do Y, DeFelice B, Meissen JK, Fiehn O (2013) LipidBlast in silico tandem mass spectrometry database for lipid identification. Nat Methods 10:755-758

13. Allen F, Pon A, Wilson M, Greiner R, Wishart D (2014) CFM-ID: a web server for annotation, spectrum prediction and metabolite identification from tandem mass spectra. Nucleic Acids Res 42:W94-99
