# Supplementary material for: Redundant roles of the phosphatidate phosphatase family in triacylglycerol synthesis in human adipocytes
Source: Diabetologia. 2016 Jun 25;59:1985–94. doi: 10.1007/s00125-016-4018-0 (PMC4969345; doi:10.1007/s00125-016-4018-0)
Supplement: Supplementary file 2 — (PDF 89.8 kb) [file 125_2016_4018_MOESM2_ESM.pdf]

ESM Table 1. *Characteristics of the cohorts for the study of the mRNA (A) and protein (B) expression.*

A.	Normoweight	Obesity	T2DM
N	17	43	11
Age (yr) [mean (SD)]	53.88 ± 15.41	57.23 ±14.08	66.09± 8.58
Sex (N, Female)	12	25	5
BMI (kg/m^2) [median (IQR)]	23.81 (1.50)	27.99 (4.60)***	28.68 (3.53)***
HOMA-IR [median (IQR)]	0.82 (1.41)	1.22 (1.20)	3.66 (21.95)*
Glucose (mM) [median (IQR)]	4.74 (0.80)	5.53 (0.64)	8.33 (3.11)***
Insulin (µIU/mL) [median (IQR)]	3.57 (5.55)	4.49 (4.20)	10.23 (17.96)
Triacylglycerol (mM) [median (IQR)]	1.04 (0.87)	1.03 (0.76)	1.66 (1.08)
NEFA (mM) [mean (SD)]	1.00 ± 0.75	0.77 ± 0.26	0.92 ± 0.41
Glycerol (mM) [median (IQR)]	177.6 (239.1)	144.3 (132.4)	301.6 (255.8)*

B.	Normoweight	Obesity	T2DM
N	9	10	9
Age (yr) [mean (SD)]	51.67 ± 3.24	52.70 ± 4.24	55.22 ± 4.84
BMI (kg/m^2) [median (IQR)]	23.30 (3.91)	33.48 (5.32)***	35.22 (6.17)***
HOMA-IR [median (IQR)]	0.63 (1.38)	2.21 (3.08)	6.92 (7.30)**
Glucose (mM) [median (IQR)]	3.78 (1.11)	4.28 (1.67)	8.56 (4.82)**
Insulin (µIU/mL) [median (IQR)]	4.00 (5.03)	12.70 (12.21)	18.73 (12.32)**
Triecylglycerol (mM) [median (IQR)]	0.81 (0.82)	1.43 (1.25)	1.72 (1.60)*

Abdominal adipose tissue (AT) was extracted from a cohort of 71 and 28 subjects for the gene (A) and protein (B) expression analysis, respectively. Participants were grouped by BMI and type 2 diabetes (T2D). Clinical and anthropometrical variables were collected. Normal distributed data are expressed as mean value (SD), and for variables with no Gaussian distribution, values are expressed as median (interquartile range, IQR). *p<0.05, **p<0.01, ***p<0.001, compared with normoweight. ANOVA, Kruskal-Wallis and Pearson χ^2 tests.
