# Supplementary material for: Redundant roles of the phosphatidate phosphatase family in triacylglycerol synthesis in human adipocytes
Source: Diabetologia. 2016 Jun 25;59:1985–94. doi: 10.1007/s00125-016-4018-0 (PMC4969345; doi:10.1007/s00125-016-4018-0)
Supplement: Supplementary file 3 — (PDF 64.4 kb) [file 125_2016_4018_MOESM3_ESM.pdf]

ESM Table 2. *Commercial reagents used in this study.*

A. ANTIBODIES

Symbol	Company		Dilution
ACTIN	Sigma-Aldrich	A2228	1/1000
CALNEXIN	Abcam	ab22595	1/3000
FABP4	Santa Cruz Biotechnology	sc-18661	1/4000
GAPDH	Santa Cruz Biotechnology	sc-32233	1/400
PPARG	Cell Signaling	2443	1/1000
SREBP1c	Santa Cruz Biotechnology	sc-8984	1/1000
TUBULIN	Sigma-Aldrich	T6557	1/3000
———————	HRP-conjugated secondary	antibodies against	———————
goat	Sigma-Aldrich	A8919	1/4000
mouse	Jackson Immunoresearch	115-035-008	1/7000
rabbit	Sigma-Aldrich	A0545	1/5000

B. HYDROLYSIS PROBES

Symbol	Gene	ID	CQ (mean ± SD) [a]
ACACA	acetyl-CoA carboxylase alpha	Hs01046047_m1	27.8 ± 0.4
AGPAT2	1-acylglycerol-3-phosphate O-acyltransferase 2	Hs00944961_m1	25.8 ± 1.6
CEBPA	CCAAT/enhancer binding protein (C/EBP), alpha	Hs00269972_s1	22.5 ± 1.3
CEBPB	CCAAT/enhancer binding protein (C/EBP), beta	Hs00270923_s1	22.5 ± 0.1[b]
CEBPD	CCAAT/enhancer binding protein (C/EBP), delta	Hs00270931_s1	29.4 ± 0.2 [b]
DGAT1	diacylglycerol acyltransferase 1	Hs00201385_m1	26.6 ± 1.3
DGAT2	diacylglycerol acyltransferase 2	Hs00261438_m1	20.9 ± 0.6
GPAT3	glycerol-3-phosphate acyltransferase 3	Hs00262010_m1	26.8 ± 1.0
LPIN1	lipin 1	Hs00299515_m1	23.2 ± 0.4
LPIN2	lipin 2	Hs00206237_m1	27.4 ± 0.3
LPIN3	lipin 3	Hs01040129_m1	25.0 ± 0.4
LPP1	phosphatidate phosphatase type 2A	Hs00170356_m1	23.0 ± 0.3
LPP2	phosphatidate phosphatase type 2C	Hs00186575_m1	28.4 ± 6.2
LPP3	phosphatidate phosphatase type 2B	Hs00170359_m1	25.7 ± 5.1
MOGAT1	monoacylglycerol O-acyltransferase 1	Hs00369695_m1	34.6 ± 1.8

MOGAT2	monoacylglycerol O-acyltransferase 2	Hs00228268_m1	34.6 ± 0.9
MOGAT3	monoacylglycerol O-acyltransferase 3	Hs00698325_m1	Non detectable
PCK1	phosphoenolpyruvate carboxykinase 1	Hs00159918_m1	20.4 ± 0.5
PPARG	peroxisome proliferator-activated receptor gamma	Hs00234592_m1	23.2 ± 0.4
PPIA	cyclophilin 1A	Hs99999904_m1	19.9 ± 0.5
SCD1	stearoyl-CoA desaturase	Hs01682761_m1	23.4 ± 0.8
SREBF1	sterol regulatory element binding transcription factor 1	Hs01088691_m1	22.1 ± 0.6

C. siRNA OLIGONUCLEOTIDES

Symbol	Company	Reference
———————— Silencer(R) Select Pre-designed siRNA ————————		
LPIN1	Ambion	S23205, S23206
LPIN2	Ambion	S18590, S18591
LPIN3	Ambion	S35072, S35073
Non-Targeting control	Ambion	No. 1
———————— On-Target Plus siRNA ————————		
LPIN1	Dharmacon	J-017427-09, -11
LPIN2	Dharmacon	J-013458-09, -11
LPIN3	Dharmacon	J-032702-07, -08
Non-Targeting control	Dharmacon	D-001810-01

[a]mean CQ ± SD from the day 10-control (non-targeting siRNA control of single knockdowns), except [b], which stands for day 4-control.
