# Supplementary material for: Redundant roles of the phosphatidate phosphatase family in triacylglycerol synthesis in human adipocytes
Source: Diabetologia. 2016 Jun 25;59:1985–94. doi: 10.1007/s00125-016-4018-0 (PMC4969345; doi:10.1007/s00125-016-4018-0)
Supplement: Supplementary file 4 — (PDF 498 kb) [file 125_2016_4018_MOESM4_ESM.pdf]

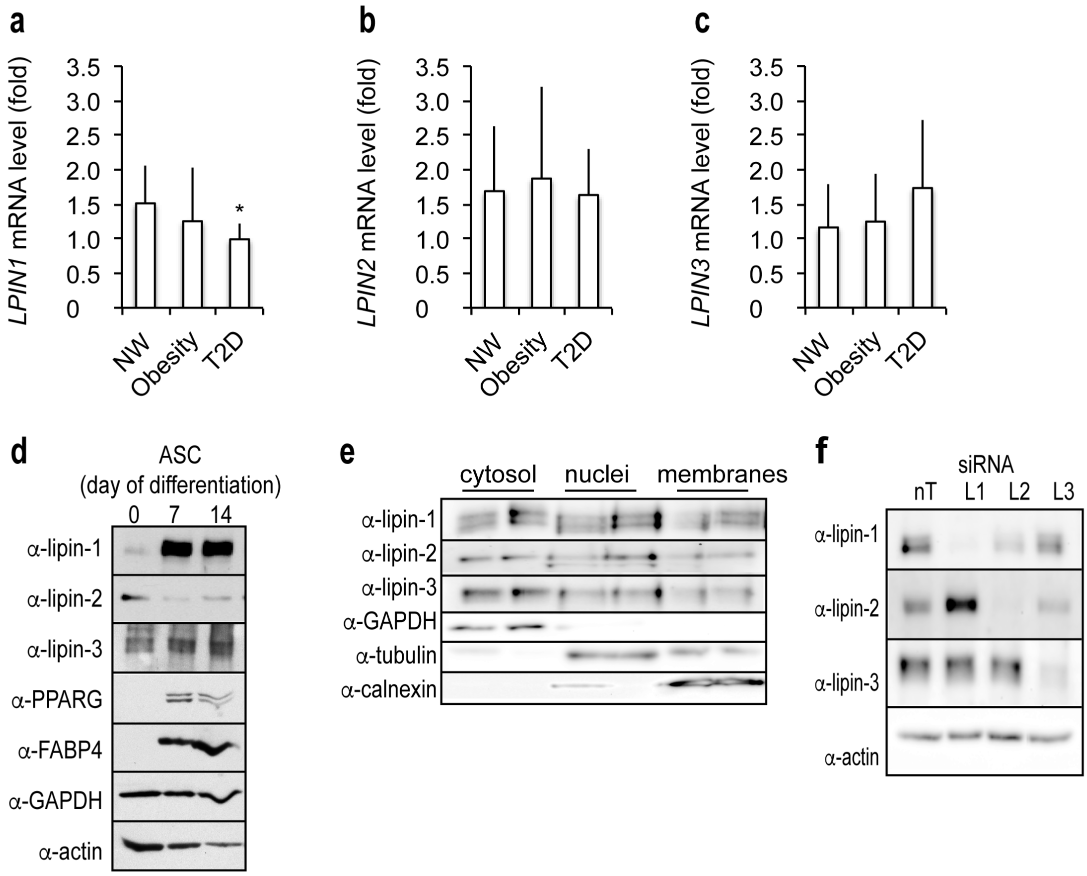

ESM Fig. 1. *Lipin expression levels in human adipocytes*. (a-c) Relative mRNA levels of the lipin family members were quantified in human abdominal visceral adipose tissue. Participants were grouped by BMI and type 2 diabetes (T2D). *, $p<0.05$ vs. normoweight; ANOVA and Kruskal-Wallis tests. (d) Protein levels of the lipin members were analysed during adipogenesis of adipose derived stem cells (ASC) from three different individuals. Protein levels of adipocyte markers (PPAR gamma and FABP4), and loading controls (glyceraldehyde-3-phosphate dehydrogenase (GAPDH), and actin), were also analysed. Portions of blots from a representative sample are shown. (e) Subcellular fractions containing cytosolic, intranuclear and membrane protein were isolated from SGBS adipocytes (day 10 after differentiation), and subcellular localization of endogenous lipin-1, -2 and -3 was assessed. Equal protein amounts from the fractions were loaded on an 8% SDS-PAGE and immunoblotted using the indicated antibodies. Markers for cytosol (GAPDH), nuclei (tubulin), and membranes (calnexin) were used. Representative portions of Western blots with duplicates are shown. (f) Single lipin-1, -2 or -3 knockdowns (L1, L2, or L3 respectively), and the non-targeting control (nT), were performed in SGBS preadipocyte cells. After induction of adipogenesis, cells were collected at day 4 and protein levels of lipins were analysed. Portions of blots from a representative sample are shown.
