# Supplementary material for: Redundant roles of the phosphatidate phosphatase family in triacylglycerol synthesis in human adipocytes
Source: Diabetologia. 2016 Jun 25;59:1985–94. doi: 10.1007/s00125-016-4018-0 (PMC4969345; doi:10.1007/s00125-016-4018-0)
Supplement: Supplementary file 5 — (PDF 318 kb) [file 125_2016_4018_MOESM5_ESM.pdf]

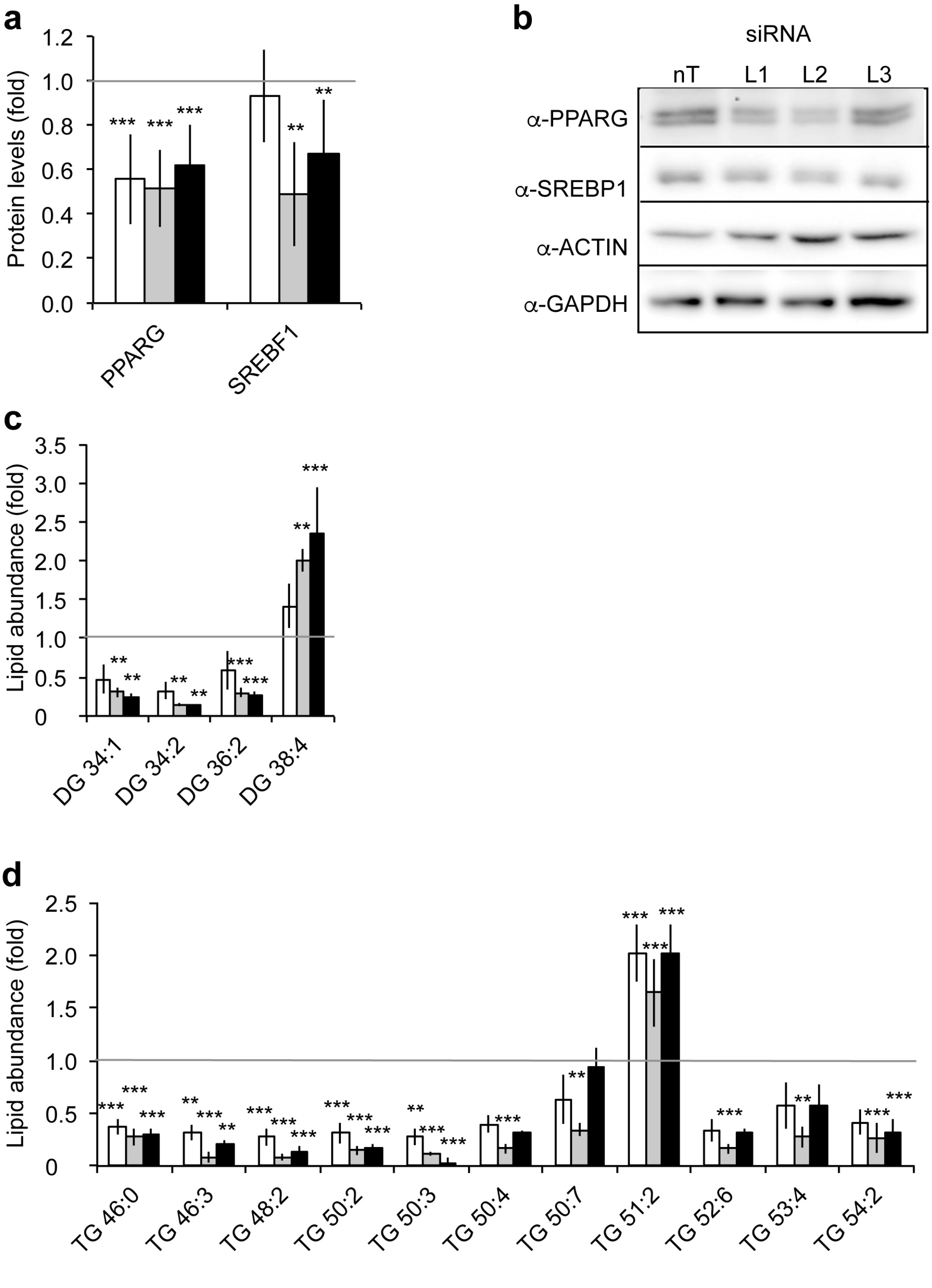

ESM Fig. 2. *The three members of the lipin family have a role in human SGBS lipogenesis in early adipogenesis stages.* Knockdowna of single lipin members were performed in SGBS preadipocyte cells. After induction of adipogenesis, cells were collected at day 4 and analysed. (a) Protein levels of transcription factors (n=7), and (b) representative portions of Western blots are shown. Abundance of significantly changed species of (c) diacylglycerol (DG) and (d) triacylglycerol (TG), (n=4). Data represent mean±SD of fold increase over non-targeting controls (set as 1). $^{**}p<0.01$, $^{***}p<0.001$, General Lineal Model Univariate test. White bars, *LPIN1* knockdown; grey bars, *LPIN2* knockdown; black bars, *LPIN3* knockdown.
