# Supplementary material for: Redundant roles of the phosphatidate phosphatase family in triacylglycerol synthesis in human adipocytes
Source: Diabetologia. 2016 Jun 25;59:1985–94. doi: 10.1007/s00125-016-4018-0 (PMC4969345; doi:10.1007/s00125-016-4018-0)
Supplement: Supplementary file 6 — (PDF 365 kb) [file 125_2016_4018_MOESM6_ESM.pdf]

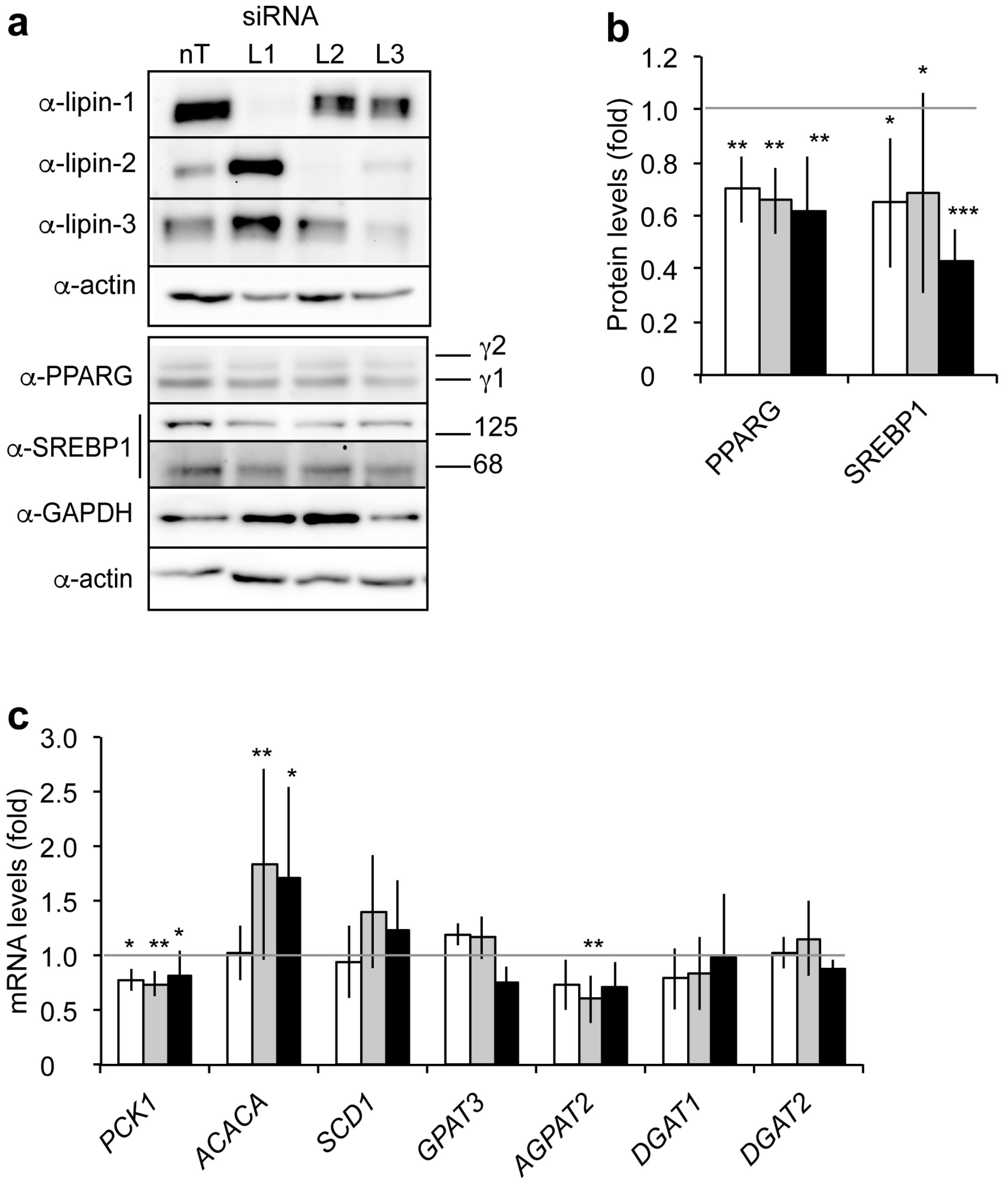

ESM Fig. 3. *Transcript and protein levels in lipin- depleted SGBS preadipocytes after fully differentiation.* Single knockdowns of lipin members were performed in SGBS preadipocyte cells. (a) At day 10 after differentiation, protein levels of the lipin family and adipocyte markers (PPAR gamma and SREBP1) were analysed. Portions of blots from a representative sample are shown. (b) Protein levels of transcription factors PPAR gamma and SREBP1 (n=5). (c) Transcript levels of genes involved in lipogenesis. Data represent mean±SD of fold increase over non-targeting controls (set as 1). *p<0.05, **p<0.01, ***p<0.001, General Lineal Model Univariate test. White bars, *LPIN1* knockdown; grey bars, *LPIN2* knockdown; black bars, *LPIN3* knockdown.
