# Supplementary material for: Redundant roles of the phosphatidate phosphatase family in triacylglycerol synthesis in human adipocytes
Source: Diabetologia. 2016 Jun 25;59:1985–94. doi: 10.1007/s00125-016-4018-0 (PMC4969345; doi:10.1007/s00125-016-4018-0)
Supplement: Supplementary file 7 — (PDF 186 kb) [file 125_2016_4018_MOESM7_ESM.pdf]

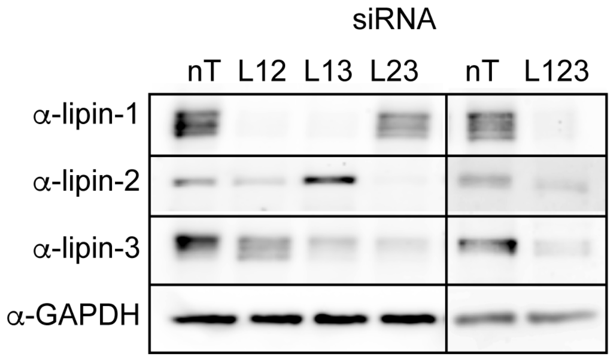

ESM Fig 4. *Combinatorial depletion of lipins in SGBS preadipocytes after fully differentiation. LPIN1* and *LPIN2* double knockdown (L12), *LPIN1* and *LPIN3* double knockdown (L13), *LPIN2* and *LPIN3* double knockdown (L23), triple knockdown (L123), and their corresponding non-targeting controls (nT), were performed in SGBS preadipocyte cells. Cells were induced to differentiate, and collected at day 10. Representative portions of Western blots are shown.
