# Supplementary material for: Redundant roles of the phosphatidate phosphatase family in triacylglycerol synthesis in human adipocytes
Source: Diabetologia. 2016 Jun 25;59:1985–94. doi: 10.1007/s00125-016-4018-0 (PMC4969345; doi:10.1007/s00125-016-4018-0)
Supplement: Supplementary file 8 — (PDF 2.26 mb) [file 125_2016_4018_MOESM8_ESM.pdf]

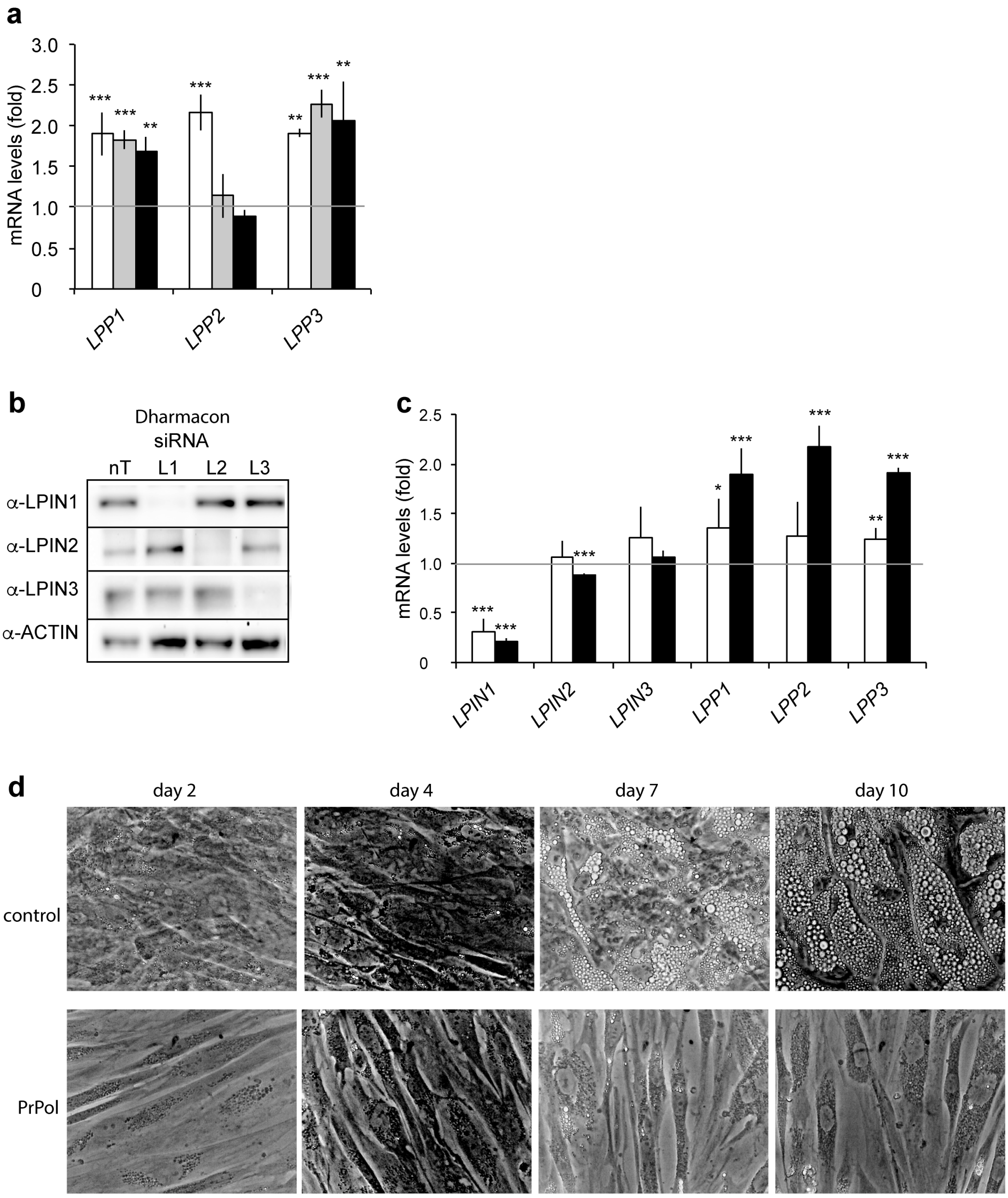

ESM Fig 5. *The LPP family is induced under conditions of repressed lipin expression.* Knockdown of single lipin members were performed in SGBS preadipocyte cells and cells were induced to differentiate. (a) Transcript levels of genes from the LPP/PAP2 family were analysed at day 4 after differentitation, relative to cyclophilin 1A and to control (n=3), General Lineal Model Univariate test. (b, c) Single lipin knockdowns, and the corresponding non-targeting control, were performed in SGBS preadipocyte cells by using siRNA from two different sources and cells were collected on day 4 after differentiation. (b) Representative portions of Western blots of extracts from cells transfected with siRNA from Dharmacon (non targeting control, nT; single *LPIN1*, L1; *LPIN2*, L2; *LPIN3*, L3 knockdowns). (c) Transcript levels of cells transfected with *LPIN1* siRNA from both Dharmacon and Ambion were analysed (n=4-5), Student T test. (d) SGBS preadipocytes were induced to differentiate in presence of 100 μmol/l propranolol (PrPol). Contrast-phase microscopy showed lipid droplet formation throughout adipogenesis is blocked in propranolol-treated cells. Data represent

mean±SD of fold increase over non-targeting controls (set as 1). **$p<0.01$, ***$p<0.001$. (a) White bars, *LPIN1* knockdown; grey bars, *LPIN2* knockdown; black bars, *LPIN3* knockdown. (c) White bars, Dharmacon siRNAs; black bars, Ambion siRNAs.
